# When informal caregivers become double victims: Development of a multilevel conceptual framework for safer home care

**DOI:** 10.1016/j.ijnsa.2026.100667

**Published:** 2026-08-25

**Authors:** Einav Srulovici, Nida Abed, Birute Mockeviciene, Ingo Neupert, Eda Atay, Betül Tosun, Kaja Põlluste, Jaakko Varpula, Mari Liukka, Sandra C Buttigieg, Cornelia Feichtinger, Paulo Sousa, Bojana Knezevic, Noemia Bessa Vilela, Ricky Bitton Cohen, Mirit Cohen, Reinhard Strametz, José Mira, Susanna Tella

**Affiliations:** a The Cheryl Spencer Department of Nursing, The University of Haifa, Israel; b Fundación para el Fomento de la Investigación Sanitaria y Biomédica de la Comunitat Valenciana (FISABIO), Valencia, Spain; c Miguel Hernández University of Elche, Elche, Spain; d Mykolas Romeris University, Vilnius, Lithuania; e RheinMain University of Applied Sciences, Wiesbaden, Germany; f Hasan Kalyoncu University, Faculty of Health Sciences, Department of Nursing, Turkey; g Hacettepe University, Faculty of Nursing, Turkey; h Institute of Clinical Medicine, University of Tartu, Estonia; i Department of Nursing Science, University of Turku, Finland; j The Wellbeing County of Ostrobothnia, Finland; k Department of Health Systems Management and Leadership, Faculty of Health Sciences, University of Malta, Malta; l College of Social Sciences, University of Birmingham, United Kingdom; m University of Applied Sciences FH Campus Wien, Vienna, Austria; n NOVA National School of Public Health, Public Health Research Centre, Comprehensive Health Research Centre, CHRC, Universidade Nova de Lisboa, Lisbon, Portugal; o University Hospital Centre Zagreb, Zagreb, Croatia; p Inštitut pravnih znanosti, Maribor, Slovenia; q Bayit Balev, Rehabilitation Center, Maccabi HealthCare Services Group, Israel; r Calite Research Group, Universidad Miguel Hernández de Elche, Spain; s Wiesbaden Institute for Healthcare Economics and Patient Safety (WiHelP), Wiesbaden Business School, RheinMain University of Applied Sciences, Wiesbaden, Germany; t Alicante-Sant Joan Health Department, Alicante, Spain; u Health Care and Social Services, LAB University of Applied Sciences, Lappeenranta, Finland

**Keywords:** Informal caregivers, Home care safety, Adverse events, Safety incidents, Double victim phenomenon, Patient safety, Conceptual framework

## Abstract

**Background:**

As healthcare increasingly shifts toward the home, the burden of care often falls on informal caregivers—family members, friends, or acquaintances—who frequently lack formal training and systemic support. This transition has intensified the risk of safety incidents in home care settings, including medication errors, falls, and other preventable harms. While patient safety researchers have traditionally focused on clinical environments, far less attention has been given to safety incidents in home care and to their cascading consequences for both care recipients and informal caregivers, who can become double victims (i.e., experiencing both the emotional impact of harm to a loved one and distress associated with their own involvement in the event).

**Purpose:**

To develop and internationally appraise a safety-oriented, multilevel explanatory framework for safer home care provided by informal caregivers, specifying how conditions across ecological levels shape everyday caregiving processes, safety incidents, and interdependent consequences for care recipients and informal caregivers.

**Methods:**

In this discursive paper, we have described the development of a conceptual framework and its international expert appraisal using a modified Delphi approach. The framework was co-developed within the BetterCare European Cooperation in Science and Technology Action CA22152 through interdisciplinary expert deliberations and a relationship-oriented narrative synthesis of literature identified in PubMed and CINAHL in September 2024. The modified Delphi process appraised the content relevance and cross-contextual interpretability of the principal framework components.

**Results:**

Thirty-six experts from 19 countries participated in the Delphi appraisal. Consensus was achieved across all framework components in the first round. The final conceptual synthesis, termed the Double Victim Ecosystem Framework for Home Care Safety (DOVE Model), integrates Donabedian’s structure–process–outcome logic with a social ecological perspective. At the micro-level, care-recipient needs, informal caregiver capacities and vulnerabilities, dyadic-relational conditions, and the home environment form an interdependent structural configuration that shapes everyday caregiving and care-coordination processes. Meso-level system capacity and professional support processes, together with macro-level policy, legal, sociocultural, and socioeconomic structures, may buffer or amplify these pathways. Safety incidents represent proximal safety outcomes that may generate cascading clinical, psychological, physical, and relational consequences, including the Double Victim Phenomenon.

**Conclusion:**

Safety in informal home care is a dynamic, multilevel capacity to anticipate and reduce avoidable harm, respond effectively when harm emerges, and support recovery and learning. The DOVE Model highlights the need for coordinated multilevel strategies to prevent safety incidents, strengthen caregiver preparedness, and provide structured support following safety incidents.

**What is already known**

• Informal caregivers provide a growing share of home care but often lack formal training and systemic support.
• Safety incidents, such as medication errors and falls, occur in home care and can harm both care recipients and informal caregivers, who can become double victims through the combined emotional impact of harm to a loved one and distress related to their own involvement in the incident.
• Patient safety research has focused largely on clinical settings, leaving home care safety insufficiently conceptualized.

**What this paper adds**

• We have proposed the Double Victim Ecosystem Framework for Home Care Safety (DOVE Model), conceptualizing safer informal home care as a dynamic ecosystem-level capacity.
• The framework makes explicit how interdependent micro-level conditions are translated into safety outcomes through everyday caregiving and care-coordination processes, while meso‑ and macro-level conditions may buffer or amplify these pathways.
• We have advanced the conceptualization of the Double Victim Phenomenon by linking safety incidents to cascading dyadic consequences and identifying multilevel leverage points for prevention, response, recovery, and learning.

## 1 Introduction

The growing shift of healthcare delivery from institutional settings to private homes has fundamentally altered the landscape of patient safety. In ageing societies, this transition has transferred complex care responsibilities into environments that are not designed as clinical settings. Consequently, informal caregivers—family members, friends, or close associates who provide unpaid or informally arranged care at home without formal healthcare qualifications or professional training—have become pivotal actors in everyday healthcare delivery (Abed et al., 2025; Roth et al., 2015).

This transformation is occurring within broader caregiving ecosystems characterized by demographic ageing and socioeconomic vulnerability. At the European level, approximately 22% of the population is aged 65 years or older, and around 21% of older adults are at risk of poverty, indicating structural pressures that shape both care needs and the resources available to meet them (Eurostat, 2025). In parallel, most day-to-day support is delivered outside formal services, with an estimated 70%–80% of care provided by unpaid informal caregivers who often dedicate 20–40 h per week to caregiving tasks (Del-Pino-Casado et al., 2021; Shang et al., 2026; Yan et al., 2024). Thus, informal caregivers play a critical role in supporting individuals with chronic illnesses, disabilities, or age-related health concerns without formal healthcare training (European Cooperation in Science and Technology [COST] Action BetterCare, 2024).

Within this context, safety incidents represent one of the most consequential threats to safety in home care. In home care, safety incidents may be conceptualised as unintended incidents occurring during care at home that cause physical or psychological harm to the care recipient or the informal caregiver and are attributable to actions or omissions in care or to failures in the organization of healthcare support—regardless of preventability (Doran et al., 2014; Mira et al., 2023; Sinn et al., 2018). Operationally, such events may result in injury, clinical deterioration, additional healthcare utilization, institutionalization, or significant caregiver distress (Abed et al., 2026).

While patient safety research has predominantly focused on institutional settings, the home represents a distinctive risk environment. Safety incidents in home care include, but are not limited to, medication errors, falls, infections, and pressure ulcers (Blais et al., 2013; Masotti et al., 2010; Schildmeijer et al., 2018). Medication errors are particularly salient in-home care, as complex regimens and administration decisions are frequently managed without professional supervision, increasing vulnerability to dosing and timing mistakes (Gil-Hernández et al., 2024). These risks emerge within caregiving environments where informal caregivers must make clinically consequential decisions with limited professional oversight and few structural safety barriers.

Importantly, harm is not limited to the care recipient. While the individual directly harmed by a safety incident—typically the care recipient—is often referred to as the first victim, Wu (2000) introduced the second victim concept to describe physicians who experience emotional, psychological, or physical distress following involvement in a medical error or adverse event. The concept was subsequently broadened and standardized through an evidence- and consensus-based definition to encompass any healthcare worker who is directly or indirectly involved in an unexpected adverse patient event, unintended healthcare error, or patient injury and is negatively affected by it (Vanhaecht et al., 2022). In home care, informal caregivers may experience a unique dual burden: they can be emotionally affected by harm to a loved one (first victim impact) while simultaneously experiencing guilt, shame, responsibility, or trauma connected to their role in the event (second victim impact). This compounded experience has been conceptualized as the Double Victim Phenomenon (Bushuven et al., 2024; Strametz, 2023).

Despite increasing recognition of safety incidents in home care, the field lacks integrative conceptual models that explain how conditions across ecological levels are translated through everyday caregiving processes into safety incidents and interdependent consequences for care recipients and informal caregivers. Against this background, the aim of this discursive paper is to develop and internationally appraise the Double Victim Ecosystem Framework for Home Care Safety (DOVE Model) as a safety-oriented, multilevel explanatory framework for safer informal home care. The framework has two interrelated functions: to explain how safety incidents and cascading dyadic consequences emerge, and to identify leverage points through which care may be made safer through prevention, timely response, recovery support, and learning. The framework was co-developed within the BetterCare COST Action CA22152 (COST Action Bettercare, 2024), and its principal components were appraised through a structured international expert consensus process using a modified Delphi approach.

## 2 Background

### 2.1 Home care safety as an ecosystem-level concern

Although the incidence and typology of safety incidents in home care have received increasing attention (Blais et al., 2013; Masotti et al., 2010; Schildmeijer et al., 2018), safety in domestic settings continues to be insufficiently conceptualized within systems-oriented frameworks that capture how individual, system, and societal factors jointly shape risk. Home environments are often compromised by inadequate training, a lack of appropriate equipment, and hazardous conditions, making them high-risk settings for both care recipients and caregivers (Gershon et al., 2008; Masotti et al., 2010; Schildmeijer et al., 2018). The growing reliance on informal caregivers, coupled with limited formal support, further exacerbates these risks, increasing the likelihood of adverse outcomes for both groups (Ngamasana et al., 2023).

Existing policy and practice guidance recognizes the vulnerability of informal caregivers and the need for structured supports. Examples include the National Institute for Health and Care Excellence guideline on supporting adult carers (National Institute for Health and Care Excellence [NICE], 2020) and the dementia guideline issued by the German Society of General Practice and Family Medicine (Deutsche Gesellschaft für Allgemeinmedizin und Familienmedizin [DEGAM], 2023). These guidelines underscore the need for comprehensive, targeted resources to support caregivers and reduce preventable harm in home settings. Nevertheless, many guidelines remain condition-specific and are largely oriented toward prevention and general caregiving support, with less explicit attention to post-adverse-event responses and caregiver psychological sequelae.

Recent analyses of home care safety further argue that safety incidents in domestic settings should not be interpreted solely as individual caregiver failures but rather as manifestations of systemic fragmentation, insufficient integration between formal and informal sectors, and structural underinvestment in community-based safety infrastructures (Vella Bonanno et al., 2025). These findings reinforce the need for a multilevel framework that conceptualizes safety in home care as an ecosystem-level phenomenon rather than a private family responsibility.

Taken together, these characteristics position home care safety as a dynamic property of the caregiving ecosystem. Clinically consequential activities are embedded within the domestic lifeworld, where care is negotiated through established relationships, everyday routines, autonomy, privacy, and variable social and material resources. Safer home care, therefore, depends on the capacity of caregiving dyads and the systems surrounding them to anticipate and reduce avoidable harm, recognize and respond to emerging risks, and support recovery following safety incidents while preserving autonomy, relational integrity, and everyday life.

### 2.2 The double victim phenomenon and caregiver psychological safety

The second victim concept has been widely used to describe clinicians who experience psychological distress following involvement in a medical error or safety incident (Wu, 2000). Extending this lens to home care is theoretically and practically warranted because informal caregivers often conduct clinically consequential tasks (e.g., medication administration, monitoring, wound care) without the structural buffers available in professional settings (Gil-Hernández et al., 2024). The Double Victim Phenomenon captures the compounded emotional and psychological burden faced by caregivers who simultaneously experience (a) the impact of harm to the care recipient and (b) the distress associated with their own involvement in the incident (Bushuven et al., 2024; Strametz, 2023). Operationally, a double victim may be conceptualised as an informal caregiver who, following a safety incident or avoidable error while caring for a loved one at home, simultaneously experiences the impact of the harm suffered by the care recipient (as the first victim) and the psychological distress resulting from their own involvement in the event (as the second victim), typically without access to formal support structures or safety barriers comparable to those available in professional care settings (Bushuven et al., 2024; Strametz, 2023).

Empirical evidence suggests that psychological sequelae among informal caregivers involved in safety incidents can be substantial. More than half of caregivers involved in safety incidents report significant psychological distress, and a considerable proportion experience prolonged emotional impact and incomplete recovery (Bushuven et al., 2024). These outcomes may compromise caregiving capacity and potentially create downstream safety risks through stress-related impairments, reduced self-efficacy, and avoidance of engagement with formal care systems.

### 2.3 Rationale for a multilevel conceptual framework

Safety incidents in home care are increasingly recognized as a significant quality and safety problem (Blais et al., 2013; Masotti et al., 2010; Schildmeijer et al., 2018). Beyond the immediate physical harms to care recipients, safety incidents can trigger downstream consequences such as clinical deterioration, increased healthcare utilization, institutionalization, and distress among caregivers (Abed et al., 2026). These outcomes highlight that the impact of safety incidents in home care extends beyond individual events and may affect the broader caregiving context.

A core challenge underlying these risks is the misalignment between the growing complexity of care delivered at home and the structural supports available to sustain safe caregiving. Home care systems are often characterized by fragmented governance, uneven regulatory protections, and insufficient integration between formal healthcare services and informal caregiving networks (Vella Bonanno et al., 2025). Such fragmentation may increase both the probability of safety incidents and the likelihood that caregivers face these events without adequate guidance, support, or recovery resources—thereby intensifying the double victim experience (Bushuven et al., 2024; Strametz, 2023).

In response to these challenges, numerous interventions have been developed to support informal caregivers. These approaches—including cognitive-behavioral, psychosocial, and technology-assisted interventions—have demonstrated beneficial effects on caregiver well-being, but these effects may be temporary and often require ongoing engagement to sustain benefits (Zhou et al., 2024). Evidence remains mixed regarding long-term effectiveness of remote supports in reducing caregiver burden and improving quality of life (Gonzalez-Fraile et al., 2021; Yu et al., 2023).

Taken together, this body of evidence suggests that interventions focused solely on individual caregivers are unlikely to be sufficient because safety in informal home care emerges through the interaction of care-recipient needs, capacities, and vulnerabilities; informal caregiver capacities, vulnerabilities, and role conditions; dyadic-relational conditions; the home environment; and the broader ecosystem in which the caregiving dyad is embedded. A multilevel framework is, therefore, required to explain how these interdependent micro-level structural conditions shape everyday caregiving and care-coordination processes and how meso‑level system capacity and professional support processes, together with macro-level policy, legal, sociocultural, and socioeconomic structures, may buffer or amplify the translation of micro-level conditions into care processes and subsequent safety outcomes (Vella Bonanno et al., 2025). By linking these cross-level conditions and processes to the emergence of safety incidents and cascading dyadic consequences, such a framework can identify safety-enabling leverage points across prevention, response, recovery, and learning.

### 2.4 Conceptual anchors: structure–process–outcome and social ecological perspectives

**Fig. 1:**
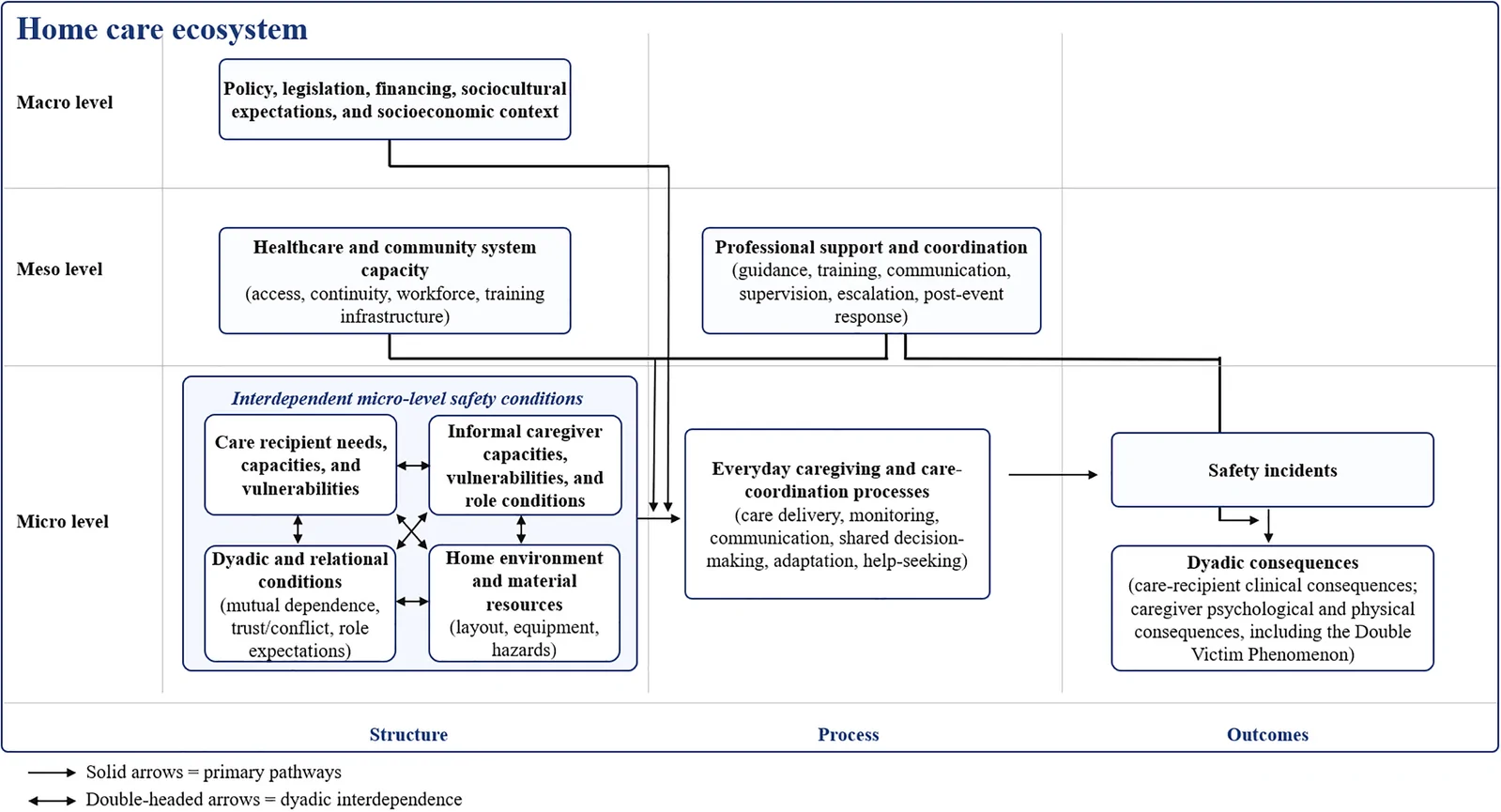
The double victim ecosystem framework for home care safety (DOVE Model).

The DOVE Model integrates Donabedian’s (1966) structure–process–outcome framework with McLeroy et al.’s (1988) Social Ecological Model within a two-axis conceptual matrix (Fig. 1). The horizontal axis represents Donabedian’s (1966) functional pathway, in which structural conditions shape care processes and, through them, safety outcomes. The vertical axis locates the conditions and processes that influence this pathway across ecological levels. McLeroy et al.’s (1988) intrapersonal, interpersonal, organizational, community, and public-policy levels are consolidated in the DOVE Model into micro-, meso‑, and macro-level domains.

The micro level contains the central structure–process–outcome pathway: interdependent conditions related to the care recipient, informal caregiver, dyadic relationship, and home environment constitute the structural domain. Everyday caregiving and care-coordination processes are conceptualized as mediating the relationship between these structural conditions and safety outcomes. The *meso* level includes both healthcare and community system capacity and professional support processes, whereas the macro level comprises broader policy, legal, sociocultural, and socioeconomic structures. Cross-level moderation links the vertical social ecological dimension to the horizontal pathway, as meso‑level structures and processes and macro-level structural conditions may buffer or amplify the translation of micro-level conditions into caregiving processes and subsequent safety outcomes.

## 3 Methods

### 3.1 Framework development sources and process

This framework was co-developed by experts participating in Working Group 3 of the BetterCare COST Action CA22152, representing Estonia, Germany, Finland, Lithuania, Spain, Turkey, and Israel. Initial framework components emerged from structured virtual and in-person deliberations synthesizing clinical, policy, and research perspectives. These deliberations focused on identifying recurrent patterns of safety risk in informal home care and on conceptualizing the multilevel mechanisms through which safety incidents unfold and affect both care recipients and caregivers.

Following the development of the preliminary model, a relationship-oriented narrative review was conducted to examine the conceptual and empirical support for each proposed pathway. The review was guided by four questions: (1) How do care-recipient needs, capacities, and vulnerabilities; informal caregiver capacities, vulnerabilities, and role conditions; dyadic-relational conditions; and home-environment conditions shape everyday caregiving and care-coordination processes? (2) How do these processes contribute to the prevention, emergence, recognition, or mitigation of safety incidents? (3) How do safety incidents generate clinical, psychological, physical, and relational consequences for both members of the caregiving dyad, including the Double Victim Phenomenon? and (4) How do meso‑level healthcare and community structures and processes and macro-level structural conditions buffer or amplify these pathways?.

The literature search was conducted in September 2024 using PubMed and CINAHL, with no restrictions on publication year. Eligible sources were English-language articles published in peer-reviewed journals. Priority was given to higher-level evidence syntheses, including systematic reviews, scoping reviews, and meta-analyses; relevant primary studies were used where review-level evidence was unavailable or insufficient to clarify a proposed relationship.

The preliminary model was divided into its proposed relationships, and each relationship was assigned to one member of the author team for focused examination. Each author identified and selected literature relevant to the assigned relationship and prepared a narrative summary of the evidence and its implications for the proposed pathway. The first author conducted the initial cross-domain synthesis by comparing the individual summaries, identifying convergent findings and conceptual links across pathways, and integrating them into a coherent multilevel framework. The resulting synthesis was circulated among all authors for critical review, refinement, and confirmation that the interpretation accurately represented the evidence identified for each relationship. Disagreements or areas requiring conceptual clarification were resolved through iterative discussion among the author group.

The development process was iterative, integrating conceptual logic of Donabedian’s (1966) structure–process–outcome framework and McLeroy et al.’s (1988) Social Ecological Model with practice-based insights from participating experts. Related components of the framework have been addressed separately in previous publications concerning informal caregiver characteristics and training needs, the Double Victim Phenomenon, and integrated approaches to safety management (Abed et al., 2025; Bushuven et al., 2024; Vella Bonanno et al., 2025). The novel contribution of the present paper lies in integrating these components within a single multilevel architecture that explicitly incorporates dyadic-relational conditions, everyday caregiving and care-coordination processes, and cross-level moderating influences.

### 3.2 International expert consensus appraisal (modified Delphi)

To obtain international expert appraisal of the content relevance and cross-contextual interpretability of the preliminary framework, a structured consensus process using a modified Delphi approach was conducted. Seventy-seven international experts from Europe, Japan, and Australia were invited to evaluate the proposed model components, of whom 36 experts from 19 countries completed the evaluation. Although participation was voluntary and the response rate was 46.8%, the breadth of geographical representation and diversity of professional expertise enhanced the credibility and cross-contextual relevance of the consensus achieved. The experts’ backgrounds included patient safety, informal caregiving, community health, health policy, and implementation science.

Participants were asked to rate the importance of each proposed domain of the conceptual framework using a 5-point Likert scale ranging from 1 (“Not important at all”) to 5 (“Extremely important”). The evaluated components addressed the broader caregiving ecosystem; policy, cultural, and legal conditions; health-system access and continuity; health-system training, guidance, and safety tools; the home environment; informal caregiver capacity; care-recipient vulnerability; high-risk care-at-home tasks as pathways to safety incidents when support is inadequate; and the Double Victim Phenomenon. A priori consensus was defined as at least 80% of experts rating a component as either 4 (“Very important”) or 5 (“Extremely important”).

## 4 Results

### 4.1 Delphi findings: consensus on framework components

All framework components achieved the predefined consensus threshold in the first Delphi round, with agreement levels ranging from 83.3% to 100% (Supplementary Material A). Consequently, additional rounds were not conducted.

This high level of agreement provided international expert support for the content relevance and cross-contextual interpretability of the principal framework components. Importantly, 94.4% endorsed high-risk home-care tasks as pathways to safety incidents when support is inadequate, supporting the inclusion of an explicit care-process domain and its dependence on the surrounding support context. Consensus also supported the relevance of the Double Victim construct within informal home care (Bushuven et al., 2024; Strametz, 2023).

### 4.2 The double victim ecosystem framework for home care safety (DOVE model)

The DOVE Model is a safety-oriented, multilevel explanatory framework that links an account of how safety incidents and cascading dyadic consequences emerge with the identification of safety-enabling leverage points. By tracing the pathway from structural conditions through everyday caregiving processes to safety outcomes, the model identifies where risk may be reduced, emerging harm recognized and addressed, and recovery supported across the home care ecosystem.

The horizontal dimension follows Donabedian’s (1966) structure–process–outcome logic. At the micro level, care-recipient needs, informal caregiver capacities and vulnerabilities, dyadic-relational conditions, and home-environment and material conditions form an interdependent structural configuration. Everyday caregiving and care-coordination processes conceptually mediate the relationship between this configuration and safety outcomes, including safety incidents and their subsequent dyadic consequences. The vertical dimension locates this pathway within the home care ecosystem. Meso-level system capacity and professional support processes and macro-level policy, legal, sociocultural, and socioeconomic structures may buffer or amplify the translation of micro-level conditions into care processes and subsequent safety outcomes.

### 4.3 Micro-level: the structure–process–outcome pathway

At the micro level, the model contains the central structure–process–outcome pathway. Interdependent structural conditions—including care-recipient needs, capacities, and vulnerabilities; informal caregiver capacities, vulnerabilities, and role conditions; dyadic-relational conditions; and the home environment—shape everyday caregiving and care-coordination processes. These processes constitute the proximal mechanism through which structural conditions are translated into safety outcomes, including safety incidents and their subsequent clinical, psychological, physical, and relational consequences for the caregiving dyad.

**Home environment and material resources.** The safety, accessibility, and adequacy of the home environment—including availability of medical equipment, layout, hygiene, and potential hazards—play a critical role in enabling or hindering safe care (Wise, 2024). Cluttered spaces, lack of mobility aids, or absence of emergency systems can elevate risk of safety incidents, particularly for care recipients with physical or cognitive impairments (Cho et al., 2016; Sama et al., 2024). These environmental conditions may not exert influence in a strictly linear or direct fashion, but rather interact dynamically with informal caregiver and care recipient vulnerabilities to contribute to safety outcomes.

**Care recipient needs, capacities, and vulnerabilities**. Certain attributes of the care recipient, such as age, cognitive impairments, physical disabilities, and comorbidities, are well-documented risk factors for safety incidents (Miller, 2012; Sears et al., 2013). At the population level, informal care recipients commonly present with chronic illness, age-related frailty and cognitive disorders, with substantial functional dependence in activities of daily living and instrumental activities—features that intensify home-care complexity and elevate safety incident risk ([OECD], 2025; Boswell et al., 2025; Zigante, 2018). Individuals with rare diseases, acute or chronic conditions, or poor health literacy face unique vulnerabilities, as they may struggle to recognize symptoms, adhere to treatments, or communicate their needs effectively (Dias et al., 2023). For instance, patients with dementia or significant mobility impairments are at a heightened risk of falls and medication errors (Masotti et al., 2010; Schildmeijer et al., 2018). These vulnerabilities may increase exposure to unintended injury or complications and shape the nature and severity of harm when safety incidents occur.

**Informal caregiver capacities, vulnerabilities, and role conditions**. Informal caregivers, who provide care without formal healthcare qualifications or professional training, may experience stress, depression, burnout, and other constraints on their capacity to sustain complex care at home (Bushuven et al., 2024; Cao et al., 2022). Caregiving capacity may also be shaped by whether assuming the role is experienced as chosen or unavoidable. Perceived lack of choice has been associated with greater physical strain, emotional stress, and adverse effects on caregiver health (Shin et al., 2023). In addition, physically demanding activities such as lifting, transferring, repositioning, and supporting mobility, may expose caregivers to musculoskeletal injury (Dimakopoulou et al., 2024).

Building on this, population-level evidence indicates that across Europe caregivers are predominantly women, a substantial proportion are aged 65 years or older, and many report chronic conditions, poorer mental health, and moderate-to-high levels of caregiving burden (Charalambous, 2023; Eurostat, 2025; Zigante, 2018). Such characteristics may directly constrain caregiver capacity, increase vulnerability to stress, and heighten the likelihood of error during complex home-care tasks (Abed et al., 2025; Ngamasana et al., 2023). These vulnerabilities are not evenly distributed. Geographic residence further stratifies caregiver risk: informal caregivers residing in rural areas are more likely to be older, have lower levels of education and income, and report poorer self-rated health compared to their urban counterparts (Boswell et al., 2025). Beyond demographic and health-related characteristics, the nature of the caregiver’s relationship to the care recipient (e.g., spouse, adult child, other relative, or friend) may shape role expectations, access to support, and coping resources (Oh et al., 2024). Informal caregivers also navigate ethical dilemmas, such as balancing autonomy with safety or administering care without formal qualifications (Riffin et al., 2021), which may amplify moral distress and contribute to Double Victim experiences (Bushuven et al., 2024).

**Dyadic and relational conditions.** The caregiver and care recipient form an interdependent dyad in which the needs, appraisals, behaviours, and outcomes of each member may influence those of the other (Lyons and Lee, 2018; Lyons et al., 2002). Dyadic-relational conditions include the history and quality of the relationship, mutual dependence, trust or conflict, power asymmetries, role expectations, and the degree of congruence between the care recipient’s preferences and the caregiver’s capacity to meet them (Li and Loke, 2014; Yoong et al., 2024). These conditions shape how care responsibilities are negotiated and whether illness management is collaborative, primarily led by one member of the dyad, or constrained by relational conflict. They also influence communication, shared decision-making, and the balance between the care recipient’s autonomy and the caregiver’s efforts to prevent harm (Miller et al., 2016; Yoong et al., 2024). Accordingly, dyadic-relational conditions are conceptualized as part of the micro-level structure, whereas communication, negotiation, and shared decision-making represent the processes through which these conditions are expressed in everyday care.

**Everyday caregiving and care-coordination processes.** Everyday caregiving and care-coordination processes conceptually mediate the relationship between micro-level structural conditions and safety outcomes. These processes include direct care delivery, such as medication administration, mobility assistance, personal care, and wound or device management; monitoring and interpretation of symptoms or clinical deterioration; communication and shared or contested decision-making within the dyad; adaptation of routines and tasks to changing needs; and help-seeking, information exchange, and coordination with formal healthcare and community services (Cao et al., 2022; Gil-Hernández et al., 2024; Kim et al., 2023; Riffin et al., 2021).

Because these processes are enacted within relationships and changing domestic circumstances, similar structural conditions may lead to different safety outcomes depending on how risks are interpreted, responsibilities are allocated, routines are adapted, uncertainty is communicated, and professional support is mobilized. Their enactment is shaped by meso‑level system capacity and professional support processes and by macro-level structural conditions, which may buffer or amplify the micro-level structure–process–outcome pathway.

**Safety outcomes: Safety incidents and dyadic consequences.** Within the DOVE Model, safety incidents represent proximal safety outcomes that may lead to harm for one or both members of the caregiving dyad. When the care recipient is harmed, they are considered the first victim (Wu, 2000). These events often have ripple effects, triggering psychological strain, guilt, and self-blame in informal caregivers. In this context, caregivers may become second victims—emotionally affected by the safety incident and by their perceived role in it. Because informal caregivers are both emotionally connected to the care recipient and directly involved in care delivery, they may experience a compounded burden. This dual burden is encapsulated in the Double Victim Phenomenon (Bushuven et al., 2024), which captures the simultaneous impact of harm to a loved one and the distress associated with the caregiver’s own involvement in the event. Consistent with this conceptualisation, empirical findings indicate that more than half of informal caregivers involved in safety incidents report significant psychological distress, with a substantial proportion experiencing prolonged emotional impact and incomplete recovery (Bushuven et al., 2024). The outcome domain, therefore, encompasses both the clinical consequences experienced by the care recipient and the psychological, physical, and relational consequences experienced by the informal caregiver and the caregiving dyad.

Within the micro-level structure–process–outcome pathway, safety incidents represent the point at which structural and relational conditions, translated through everyday caregiving and care-coordination processes, become manifest as harm and cascading dyadic consequences. By integrating care-recipient harm and caregiver consequences within a shared outcome domain, the framework identifies leverage points for prevention, timely response, recovery support, and mitigation of caregiver distress (Abed et al., 2026; Vella Bonanno et al., 2025).

### 4.4 Meso-level: healthcare and community structures and processes

At the *meso* level, healthcare and community systems include both structural capacity and processes enacted at the interface between informal and formal care. This level encompasses the infrastructure, resources, and support mechanisms provided by formal healthcare and community services, which influence both the likelihood of safety incidents and their consequences for informal caregivers. Structural components include service availability and accessibility, workforce capacity, continuity mechanisms, training infrastructure, and community resources, whereas process components include the actual provision of guidance, communication, coordination, supervision, escalation, and post-event support**.** A key aspect of this support is recognizing the informal caregiver as a key system navigator and advocate for the care recipient. Given that caregivers often possess long-term, detailed knowledge about the care recipient’s needs and preferences—knowledge that formal providers may lack (Kim et al., 2023)—it is essential that healthcare systems integrate this expertise into care strategies to ensure quality and safety in home settings. Together, meso‑level structures and processes may buffer or amplify the translation of micro-level conditions into everyday caregiving processes and subsequent safety outcomes.

**Access to formal care.** Access to formal home care services plays a critical role in reducing safety incidents by providing professional support and alleviating some caregiving burdens. Researchers have reported that integrating formal care into home care settings may reduce risks associated with medication mismanagement and improper use of medical devices (Lyu et al., 2024). Conversely, inadequate access to these services increases the strain on informal caregivers, making them more susceptible to safety incidents (Lindt et al., 2020).

**Training and educational resources.** Informal caregivers often lack the necessary training to safely manage complex care needs. Formal healthcare providers can enhance caregiver competencies through targeted training programs and educational resources, reducing the occurrence of safety incidents (Juin, 2019). Regular supervision and guidance from healthcare professionals have been shown to improve caregivers’ decision-making abilities, particularly in high-risk tasks, such as medication administration (Cao et al., 2022). Within the model, the availability of training infrastructure, educational resources, and qualified personnel represents meso‑level structural capacity. The actual provision of timely, tailored, and comprehensible training, together with assessment of understanding and follow-up, constitutes a meso‑level professional support process.

**Professional support and coordination processes.** Meso-level processes also include ongoing communication between informal caregivers and professionals, information exchange across services, supervision of complex or high-risk tasks, follow-up, and clear pathways for seeking guidance or escalating concerns. These processes are particularly important when caregivers encounter uncertainty, changes in the care recipient’s condition, or emerging safety risks. Following a safety incident, they may also include clinical reassessment, non-punitive communication, psychological support, and coordination of changes to the caregiving arrangement. Through these processes, available healthcare and community resources are translated into support that can be accessed and used in everyday care (Kim et al., 2023; Riffin et al., 2021).

**Patient safety guidelines for care at home.** Several existing guidelines provide practical, actionable frameworks for caregivers and healthcare professionals in home care. Most focus on condition-specific support, such as dementia or stroke caregiving (De Coninck et al., 2017; DEGAM, 2023; NICE, 2020; Scottish Intercollegiate Guidelines Network [SIGN], 2023; Stroke Foundation, 2017). However, the NICE guidelines stand out for addressing caregiver support across a wide range of needs rather than being limited to a single condition. These guidelines comprehensively cover general caregiver support and safe caregiving practices, making them applicable across diverse caregiving contexts. Notably, existing guidelines do not address post-adverse-event actions, highlighting a gap in guidance on how caregivers should respond to safety incidents in home settings (Vella Bonanno et al., 2025). This gap concerns not only the availability of guidance as a structural resource but also the absence of clearly defined response and support processes following an incident.

**Support interventions.** Recent evidence suggests that interventions for informal caregivers can reduce caregiver burden, depression, and stress and improve quality of life, with multicomponent approaches combining psychoeducation, cognitive-behavioral techniques, coping strategies, and social support showing particularly promising effects across multiple caregiver outcomes (Barrero-Mejias et al., 2024; Wang et al., 2024). These findings support the value of multifaceted approaches capable of addressing the diverse psychological, social, and practical needs associated with caregiving. Cognitive-behavioral and psychosocial interventions, including mindfulness and group therapies, show positive but often temporary effects on caregiver well-being, highlighting the need for sustained and diversified support strategies (Boele et al., 2019; Mackenzie, 2021; Treanor et al., 2019). Although remote interventions have been explored, particularly in dementia care, evidence remains inconclusive regarding their long-term effectiveness in reducing caregiver burden or improving quality of life (Gonzalez-Fraile et al., 2021; Yu et al., 2023). Additionally, technology-based and e-health interventions offer promising strategies for alleviating caregiver burden by providing accessible and tailored support (Li et al., 2022; Zhou et al., 2024). However, while these innovations represent progress, comprehensive evidence on their long-term benefits is still lacking. Further research is necessary to develop sustained, evidence-based interventions that enhance both caregiving safety and caregiver well-being.

Taken together, meso‑level structures and processes influence what support is available, whether it reaches the caregiving dyad, and how effectively it is enacted during routine care, emerging risk, and post-event response. Their cross-level moderating influence may buffer or amplify the effects of micro-level structural conditions on everyday caregiving processes and subsequent safety outcomes.

### 4.5 Macro-Level: policy, legal, sociocultural, and socioeconomic structures

The macro level encompasses societal, cultural, and regulatory factors that shape the broader caregiving environment. Within the DOVE Model, these factors are conceptualized primarily as structural conditions that determine the distribution of resources, rights, responsibilities, and expectations surrounding informal home care. Recent researchers have highlighted that home care systems across Europe are characterized by fragmented governance structures and uneven regulatory protections, often leaving informal caregivers operating in ambiguous legal and institutional spaces (Vella Bonanno et al., 2025). Such structural fragmentation may amplify both safety incident risk and the psychological consequences that follow.

**National healthcare policies.** Countries with robust caregiver support systems, such as financial compensation, respite care, and accessible formal healthcare services, may provide stronger structural conditions for safer home care (Juin, 2019). National policies that provide financial aid and training for informal caregivers may reduce caregiver burden and strengthen caregivers’ capacity to manage complex care responsibilities (Cartaxo et al., 2023).

**Cultural expectations and health beliefs**. Cultural norms, values, and health beliefs strongly influence caregiving experiences. In cultures where caregiving is viewed as a familial duty without external support, caregivers experience greater emotional strain, increasing the risk of safety incidents (Cao et al., 2022). Additionally, cultural perceptions of caregiving roles and responsibilities affect how caregivers interact with healthcare systems and approach care tasks (Agyemang-Duah and Rosenberg, 2023). In societies where caregiving is framed as a shared community responsibility and external support is more readily mobilized, the burden placed on individual caregivers may be reduced, with potential benefits for both caregivers and care recipients (Cao et al., 2022).

**Legal frameworks**. Legislation constitutes a macro-level structural condition that may exert a cross-level moderating influence by determining whether caregivers are legally recognized, protected, and supported in their roles. When laws provide clear definitions of caregiving responsibilities, access to paid leave, financial compensation, and legal standing in decision-making, they reduce the vulnerability of caregivers and buffer the emotional and logistical consequences of safety incidents. While some national and state-level policies provide access to financial support, respite, or workplace protections, many lack legal clarity regarding caregivers’ rights and responsibilities, are inconsistently implemented, or fail to reach vulnerable groups (Green et al., 2024). Moreover, the framing of caregiving as “informal” often leads to its exclusion from formal care systems, weakening legislative action and system responsiveness (Kemp, 2015). As such, caregiver legislation can only function as an effective moderator when it moves beyond symbolic recognition and establishes enforceable, equitable, and integrated protections within health and social policy frameworks (Green et al., 2024; Vella Bonanno et al., 2025).

**Socioeconomic conditions.** Socioeconomic conditions shape the resources available to caregiving dyads and their capacity to obtain formal support, equipment, respite, and timely professional assistance. Household income, employment flexibility, geographic location, and the affordability and availability of services may determine whether caregivers can reduce competing demands or obtain help with complex care responsibilities. These conditions contribute to unequal exposure to home care safety risks and to variation in the buffers available across caregiving contexts (Boswell et al., 2025; Eurostat, 2025; Zigante, 2018).

## 5 Discussion

In this discursive paper, we have advanced the Double Victim Ecosystem Framework for Home Care Safety (DOVE Model), a multilevel conceptual framework that positions safety in informal home care as an ecosystem-level phenomenon shaped by interacting micro-, meso‑, and macro-level determinants. The model integrates an explanation of how safety incidents and cascading dyadic consequences emerge with the identification of points at which safety may be strengthened. By linking a micro-level structure–process–outcome pathway with cross-level ecological influences, the DOVE Model provides a conceptual bridge between patient safety research and the growing global reliance on informal caregiving.

Within this framework, home care safety is understood as an ongoing and context-sensitive accomplishment. It depends on the capacity of caregiving dyads and the systems surrounding them to anticipate and reduce avoidable harm, recognize and respond to emerging risks, and support recovery following safety incidents. Because these capacities are enacted within the domestic lifeworld, they must coexist with autonomy, privacy, established relationships, and the continuity of everyday life. This conceptualization establishes safer home care as an actionable goal requiring continuous adaptation across the caregiving ecosystem.

The modified Delphi consensus provides international expert support for the content relevance of the principal conditions, processes, and consequences incorporated into the framework. Experts endorsed care-recipient vulnerability, informal caregiver capacity, the home environment, healthcare-system access and support, broader ecosystem and policy conditions, high-risk home-care tasks as pathways to safety incidents when support is inadequate, and the Double Victim construct. The detailed structure–process–outcome ordering, the explicit dyadic-relational domain, and the proposed mediating and cross-level moderating relationships were further articulated through the integrative conceptual synthesis and now require empirical testing.

Conceptually, the framework extends patient safety thinking beyond institutional settings by integrating caregiver psychological safety into the same explanatory architecture as the emergence of safety incidents. This integration is important because it highlights how caregiver distress is not merely a downstream “burden” outcome but an integral component of the dyadic consequences of safety incidents. By locating clinical harm to the care recipient and psychological, physical, and relational consequences for the caregiver within the same outcome domain, the framework broadens the scope of home care safety beyond patient outcomes alone (Bushuven et al., 2024; Kim et al., 2023).

## 6 Safety-enabling implications

The framework also strengthens the field by explicitly situating safety incidents and informal caregiver outcomes within broader structural and systemic conditions. Recent researchers have argued that home care safety failures often reflect system fragmentation and underinvestment provide strong justification for ecosystem framing (Vella Bonanno et al., 2025). In this sense, the framework functions as a scaffold for identifying where responsibility and accountability should be located: not solely with caregivers but across health systems and policy domains. This is aligned with the premise that home care safety cannot be sustained through individual coping interventions alone. Instead, safety requires coordinated cross-level strategies: improving home environments and caregiver capacity and assessing dyadic-relational conditions at the micro level (Cho et al., 2016; Wise, 2024); ensuring accessible and integrated professional support, guidance, coordination, escalation pathways, and post-event response processes at the *meso* level (Lindt et al., 2020; Lyu et al., 2024); and strengthening policy, financial, socioeconomic, and legal protections at the macro level to reduce inequities and ambiguity in caregiver roles and rights (Green et al., 2024; Kemp, 2015).

From a practice and policy perspective, the framework highlights an actionable gap in existing guidance: while prevention-oriented guidelines and condition-specific pathways are increasingly available (DEGAM, 2023; NICE, 2020; SIGN, 2023), post-adverse-event guidance for informal caregivers remains largely absent. This omission is particularly consequential given the salience of the Double Victim Phenomenon (Bushuven et al., 2024; Strametz, 2023). Accordingly, future guideline development should incorporate clear, accessible protocols for post-event response, caregiver psychological support, and pathways for rapid linkage to formal services. Together, these strategies extend safety across anticipation and prevention, recognition and response, mitigation of clinical and psychological consequences, and recovery and learning.

The framework is intentionally designed to be transferable across systems, yet it is also sensitive to cross-national variation. Macro-level moderators—policy, law, and cultural expectations—may shape not only the risk of safety incidents but also the likelihood that informal caregivers disclose events, seek help, and access supports (Agyemang-Duah and Rosenberg, 2023; Cao et al., 2022; Green et al., 2024). This implies that implementation strategies should be context-sensitive, with attention to how “informality” is constructed and operationalized within national systems (Kemp, 2015; Vella Bonanno et al., 2025). The availability and influence of these moderators may differ substantially between systems with universal coverage and those in which access depends more strongly on insurance arrangements, geography, or household resources.

Finally, while the framework is conceptually robust and supported by international consensus on its principal components, its practical value now depends on empirical testing. Longitudinal studies, implementation research, and policy analyses are needed to examine how the proposed pathways operate across diverse caregiving dyads and systems and to identify which meso‑ and macro-level interventions most effectively prevent safety incidents and buffer caregiver psychological harm. Empirical work should also explore how interventions interact across levels—for example, whether training effects depend on system accessibility, or whether legal protections buffer distress following safety incidents—thereby operationalizing the ecosystem logic that underpins the model. In particular, researchers should test the proposed process mediation at the micro level and the cross-level moderating influences of meso‑ and macro-level conditions.

## 7 Limitations and future research

Several limitations should be considered. First, the literature synthesis was narrative and relationship-oriented rather than systematic. This approach enabled focused examination of the individual pathways and their subsequent interdisciplinary integration, but relevant evidence may have been missed. Future systematic, realist, or mechanism-focused reviews could provide a more comprehensive and reproducible assessment of the proposed relationships.

Second, the modified Delphi included experts with diverse professional backgrounds from 19 countries but did not include informal caregivers or care recipients. Future refinement should, therefore, incorporate co-design with care recipients and informal caregivers from diverse cultural, socioeconomic, and care contexts.

Third, the framework was developed primarily within a European collaboration. Its major domains—caregiver capacity, access to services, caregiver support, legal recognition, and formal–informal care integration—are relevant across healthcare systems, but their operational meaning and relative influence are likely to vary. Differences between systems with universal coverage and those in which access depends more strongly on insurance status, geography, or household resources may substantially alter the available safety buffers. Cross-national research is needed to examine these contextual variations.

Finally, the DOVE Model is a conceptual framework and its proposed pathways have not yet been empirically tested. The model provides a structured basis for generating hypotheses and selecting intervention targets, but longitudinal dyadic studies, multilevel analyses, and implementation research are required to examine temporal ordering, process mediation, and cross-level moderation across diverse home care contexts.

## 8 Conclusion

This multilevel framework offers a theory-grounded and practice-oriented model for understanding and addressing safety risks in home care. The DOVE Model conceptualizes safety as an ecosystem-level phenomenon produced through interactions among interdependent care-recipient, informal caregiver, dyadic-relational, and home-environment conditions; everyday caregiving and care-coordination processes; meso‑level healthcare and community structures and processes; and macro-level policy, legal, sociocultural, and socioeconomic structures. The modified Delphi consensus provides international expert support for the content relevance of the framework’s principal components, prior to empirical field testing.

By introducing the Double Victim Phenomenon as a central construct, we have highlighted a critical yet under-recognized consequence of safety incidents in home care and extended patient safety discourse beyond institutional environments. Ensuring safer care at home requires more than isolated caregiver-focused interventions; it demands coordinated strategies that strengthen structural conditions, improve care processes, and mitigate emotional and psychological harm to both care recipients and informal caregivers.

In alignment with the goals of the BetterCare COST Action CA22152, this framework promotes a proactive and inclusive vision of home care safety. Although developed in a European context, its principles may extend across diverse healthcare systems, as access to care, caregiver support, legal recognition, and formal–informal care integration are incorporated as system-level conditions. By conceptualizing safety as a shared, ecosystem-level responsibility, the framework supports efforts to build more resilient, equitable, and safer caregiving environments and responds to emerging calls for clearer accountability structures and stronger integration between formal and informal care systems (Vella Bonanno et al., 2025). The framework, thereby, links an explanation of how harm emerges with leverage points for prevention, response, recovery, and learning across the home care ecosystem.

## AI usage disclosure statement

The authors used ChatGPT solely for proofreading and language refinement of the manuscript. The AI tool was not used to generate scientific content, develop ideas, conduct analyses, interpret findings, or make substantive editorial decisions. All intellectual content, analyses, interpretations, and conclusions are the responsibility of the authors.

## Ethics declaration

The authors have NOT obtained informed consent from participants or their legal representatives. Formal informed consent was not required under applicable institutional guidelines. The Delphi component involved voluntary expert appraisal of the conceptual framework and did not involve patients or caregivers, clinical interventions, or the collection of identifiable or sensitive personal data.

This study was performed in compliance with relevant laws, regulatory frameworks and guidelines where the research took place. This study was conducted in accordance with CREDES (Conducting and REporting of DElphi Studies). Ethics committee approval was not required under relevant laws and institutional guidelines. The Delphi component involved expert appraisal of the conceptual framework rather than research involving patients or caregivers and did not include clinical interventions or the collection of identifiable or sensitive personal data. Participation was voluntary.

## Funding

This article is based upon work from BetterCare, CA22152, supported by E-COST (European Cooperation in Science and Technology).

## CRediT authorship contribution statement

**Einav Srulovici:** Writing – original draft, Visualization, Validation, Project administration, Methodology, Investigation, Formal analysis, Conceptualization. **Nida Abed:** Writing – review & editing, Visualization, Project administration, Methodology, Investigation, Formal analysis, Conceptualization. **Birute Mockeviciene:** Writing – review & editing, Visualization, Investigation, Conceptualization. **Ingo Neupert:** Writing – review & editing, Visualization, Investigation, Conceptualization. **Eda Atay:** Writing – review & editing, Visualization, Investigation, Conceptualization. **Betül Tosun:** Writing – review & editing, Visualization, Investigation, Conceptualization. **Kaja Põlluste:** Writing – review & editing, Visualization, Conceptualization. **Jaakko Varpula:** Writing – review & editing, Visualization, Investigation, Conceptualization. **Mari Liukka:** Writing – review & editing, Visualization, Investigation, Conceptualization. **Sandra C Buttigieg:** Writing – review & editing, Visualization, Conceptualization. **Cornelia Feichtinger:** Writing – review & editing, Visualization, Conceptualization. **Paulo Sousa:** Writing – review & editing, Visualization, Conceptualization. **Bojana Knezevic:** Writing – review & editing, Visualization, Conceptualization. **Noemia Bessa Vilela:** Writing – review & editing, Visualization, Conceptualization. **Ricky Bitton Cohen:** Writing – review & editing, Visualization, Conceptualization. **Mirit Cohen:** Writing – review & editing, Visualization, Conceptualization. **Reinhard Strametz:** Writing – review & editing, Visualization, Project administration, Methodology, Conceptualization. **José Mira:** Writing – review & editing, Visualization, Validation, Project administration, Methodology, Conceptualization. **Susanna Tella:** Writing – review & editing, Visualization, Validation, Project administration, Methodology, Investigation, Conceptualization.

## Declaration of competing interest

The authors declare the following financial interests/personal relationships which may be considered as potential competing interests:

All authors reports administrative support and travel were provided by European Cooperation in Science and Technology. If there are other authors, they declare that they have no known competing financial interests or personal relationships that could have appeared to influence the work reported in this paper.
