# Supplementary material for: When informal caregivers become double victims: Development of a multilevel conceptual framework for safer home care
Source: Int J Nurs Stud Adv. 2026 Aug 25;11:100667. doi: 10.1016/j.ijnsa.2026.100667 (PMC13579581; doi:10.1016/j.ijnsa.2026.100667)
Supplement: Supplementary file 1 [file mmc1.docx]

**Supplementary Material A.** Modified Delphi results

| **Conceptual framework domains** | **%  Agreement** | **Mean (±SD)** |
| --- | --- | --- |
| **Ecosystem characteristics.** In your opinion, how important is the broader caregiving ecosystem (e.g., social support networks, community resources, socioeconomic conditions) in influencing safety in informal home care | 100% | 4.75 (0.44) |
| **Ecosystem characteristics** (policy/culture/legal). In your opinion, how important are policies, cultural expectations, and legal protections for informal caregiving in shaping adverse event and caregiver harm? | 83.3% | 4.28 (0.74) |
| **Health system.** In your opinion, how important is timely access to and continuity of professional support (health and home-care services) in reducing adverse events in informal home care? | 97.2% | 4.81 (0.47) |
| **Health system** (training/tools/support). In your opinion, how important are health-system supports (caregiver training, clear guidance, usable safety tools) for safe informal home care? | 100% | 4.83 (0.38) |
| **Home environment characteristics.** In your opinion, how important are home environment safety and adequacy factors (e.g., hazards, accessibility, hygiene, equipment/supplies, emergency systems) in preventing adverse events? | 97.2% | 4.72 (0.51) |
| **Informal caregiver characteristics.** In your opinion, how important is caregiver capacity (e.g., physical/mental health, stress/burnout, coping resources, moral distress) in influencing safety outcomes? | 100% | 4.72 (0.45) |
| **Care recipient characteristics.** In your opinion, how important is care-recipient vulnerability (e.g., cognitive impairment, multimorbidity, mobility limitations, low health literacy) as a driver of adverse event at home? | 86.1% | 4.44 (0.73) |
| **Care at home.** In your opinion, how important are high risk home-care tasks (e.g., medication management, device use, wound care, infection prevention) as pathways to adverse events when support is inadequate? | 94.4% | 4.64 (0.59) |
| **Double victim.** In your opinion, how important is it to measure how often informal caregivers experience double victimization? | 83.3% | 4.31 (1.04) |
